# Impact of smoking on resin bonded restorations: A narrative review

**DOI:** 10.18332/tid/188114

**Published:** 2024-05-29

**Authors:** Meshal Alonazi

**Affiliations:** 1 Department of Restorative Dental Sciences, College of Dentistry, Jouf University, Sakaka, Kingdom of Saudi Arabia

**Keywords:** smoking, dental aesthetics, resin-based restorations, composite materials, color stability

## Abstract

**INTRODUCTION:**

Smoking is a common risk factor for multiple diseases, including diabetes mellitus, cardiovascular diseases, pulmonary diseases, and different cancers. It is an established cause of multiple oral health conditions, in addition to adversely impairing the appearance of oral tissues. Smoking has also been identified to impact dental restorations’ functional and esthetic aspects. This narrative review is focused on the significance of the esthetic consequences of smoking on resin-based dental restoration. It provides an understanding of the challenges dental professionals and patients face.

**METHODS:**

A literature search was conducted to identify studies on the impact of smoking exposure on the esthetic appearance of composite restorations. The inclusion criteria were met by five studies selected for analysis.

**RESULTS:**

The studies revealed that conventional cigarettes cause more noticeable color changes than electronic cigarettes. Additionally, the choice of composite material significantly influences the color stability of dental restorations. Exposure to both smoking and staining agents could worsen the color alterations in dental restorations. Smokers may benefit from dental ceramics, which offer better aesthetics. Quantitative insights into these effects can be gained by incorporating delta E (ΔE) values and fluorescence intensity in esthetic evaluations.

**CONCLUSIONS:**

The studies provide valuable insights into the multiple factors that can impact the aesthetics of dental restorations. Dental professionals should take into account the smoking type, composite material, and patient habits when planning treatments. Utilizing ΔE values and fluorescence intensity in esthetic evaluations can provide a more comprehensive understanding. Future research should focus on longitudinal studies, alternative materials, and individual patient profiles.

## INTRODUCTION

According to the location of the stain, tooth discoloration has been classified histologically into two categories: extrinsic (occurs outside the tooth substance and lies on the tooth surface or in the acquired pellicle due to presence of organic chromogens or due to tobacco smoking and chewing, consumption of tea and coffee, use of chlorhexidine, etc.) and intrinsic (occurs after a change in the structural composition or thickness of the dental hard tissues due to some metabolic diseases, local and systemic factors)^1^.

Smoking has long been recognized as a major cause of various health problems, including respiratory and cardiovascular diseases^2^. However, its harmful effects are not limited to general health but also extend to oral health and esthetics, where the effects can be particularly pronounced^3^.

Composite resins, a type of resin-based dental restoration, have become the preferred choice for dental treatments due to their versatility, durability, and esthetic properties^4^. These materials are used for various applications like filling cavities, repairing damaged teeth, and creating veneers^5^. The color stability and overall appearance of dental restorations are essential factors that impact patient satisfaction and self-esteem^6^. Patients expect dental treatments to restore their oral health and enhance their smiles. Achieving a harmonious blend between dental restorations and natural teeth is a crucial goal of esthetic dentistry^7^.

Dental aesthetics has distinct challenges, which include smoking, and it does not matter if one smokes conventional cigarettes or electronic cigarettes (E-cigarettes). This particular habit’s effects on the aesthetic and durability characteristics of resin-based dental materials are just beginning to be researched. In this case, smoking leads to the prominent aesthetic effect of discolorations. Conventional cigarette smoke contains tar and other combustion byproducts deposited on dental restorations, causing discoloring and staining effects^8-10^.

Despite being promoted as a safer alternative to conventional cigarettes, e-cigarettes are not without aesthetic drawbacks. The aerosol produced by these devices may contain nicotine, which can interact with components of dental materials, causing discoloration and affecting the clarity of restorations^11^. The choice of composite material used for dental restorations is another factor that can influence their aesthetic stability. Different composite materials vary in their susceptibility to color changes, making material selection a crucial consideration when planning treatments for patients who smoke^12^.

Dental restorations can be compromised by smoking as well as the consumption of staining agents such as coffee, tea, red wine, and cola-based soft drinks. This can pose a challenge for patients who enjoy these beverages and smoke^13^. Dental professionals must balance the need to provide patients with aesthetically pleasing and durable restorations with the impact of smoking and staining agents^14^. Practical guidance on managing these esthetic challenges is needed. Dental practitioners need to navigate the nuances of material selection, smoking cessation counseling, and maintenance recommendations to ensure the longevity and esthetic appeal of dental restorations in a smoking population.

This narrative review seeks to explore and elucidate the inquiries surrounding the esthetic consequences of smoking on resin-based dental restorations. Through a thorough examination and synthesis of recent studies, the aim is to equip dental practitioners with insights into the subtleties of these esthetic issues. This understanding will empower professionals to offer personalized advice and treatment strategies, enhancing the likelihood of successful outcomes and patient contentment. In the subsequent sections, the approaches and findings of chosen studies will be meticulously examined, presenting a thorough panorama of the subject and illuminating the strategies for upholding dental aesthetics in individuals who smoke.

## THE AESTHETIC IMPACT OF SMOKING ON RESIN-BASED DENTAL RESTORATIONS

This review investigates the aesthetic impact of smoking on resin-based dental restorations.

PICO framework was used to frame the research question, which focused on the problem area, intervention, comparison, and outcome. The following question was used:

‘In adult smokers, does smoking have a more significant esthetic impact on resin-based dental restorations compared to non-smokers, as measured by color change and staining?’.

### Search strategy

A systematic search was conducted in the PubMed database using the following search string: [‘aesthetic impact’ OR ‘esthetics’ OR ‘color change’ OR ‘visual changes’] AND [‘smoking’ OR ‘cigarette smoking’ OR ‘tobacco smoking’ OR ‘nicotine use’ OR ‘Electronic Nicotine Delivery Systems’] AND [‘resin-based restorations’ OR ‘composite resins’ OR ‘dental composites’ OR ‘dental restorations’]. The initial search returned 122 results.

### Filtering results

The search was refined to include only articles in English, resulting in 119 articles.

Articles published within the past ten years were considered, leading to 68 articles. Focusing on the last ten years allowed to identify the most recent developments, trends, and advancements in the research area. This also ensured that the review reflects current knowledge and is relevant to contemporary issues and practices.

### Inclusion criteria

- Studies that focus on composite restorations.
- Studies involving exposure to cigarette or e-cigarette smoke.
- Experimental studies, randomized controlled trials, or case-control studies.

### Exclusion criteria

- Studies related to CAD/CAM restorations only.
- Review articles or systematic reviews.
- Studies not available in full text.

**Figure 1:**
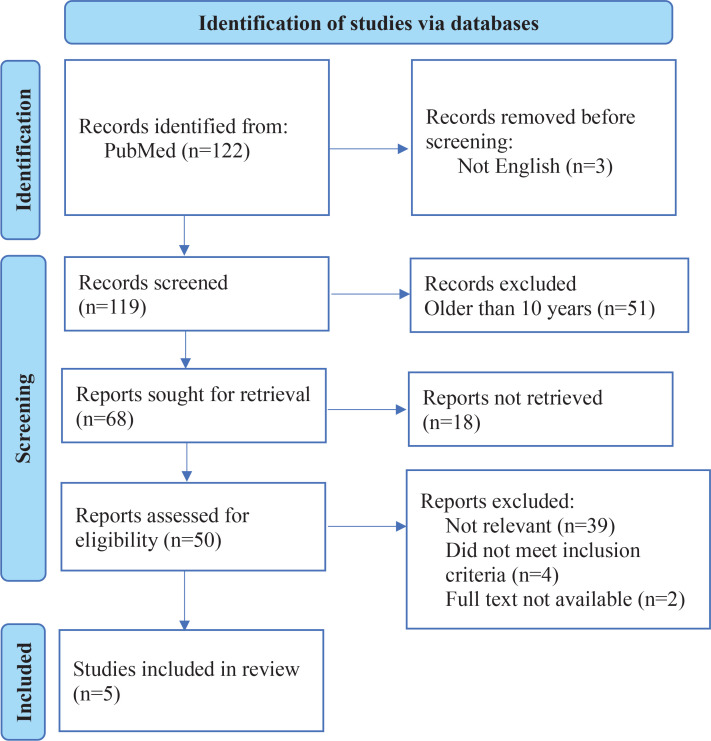
Flowchart of the search strategy

The systematic search strategy was meticulously designed to identify and evaluate studies that specifically address the impact of smoking on resin-based dental restorations. The review aimed to ensure the selection of high-quality studies aligned with the research question while adhering to the defined inclusion and exclusion criteria. After reviewing titles, 11 articles were selected for further consideration. Abstracts were screened, and the inclusion and exclusion criteria were applied, ultimately selecting seven articles for full-text review. After applying the criteria, five studies were chosen for inclusion in the review. (Figure 1, Tables 1–3)^14-18^.

**Table 1:** Summary of the methodologies and critical findings of the selected studies investigating the aesthetic impact of smoking on resin-based dental restorations

*Study title*	*Study design*	*Sample size and material*	*Type of cigarette*	*Key findings*	*Clinical implications*
Effect of conventional and electronic cigarettes smoking on the color stability and clarity of tooth-colored restorative materials: an *in vitro* analysis^15^ (2021)	An *in vitro* analysis	120 specimens - Nano-hybrid Filtek Z250 XT and Micro-hybrid Filtek Z250	Conventional and electronic cigarettes	Conventional cigarettes have a more pronounced effect on color change than electronic cigarettes. The micro-hybrid composite showed better color stability. Translucency decreased in all groups.	Dental material selection and smoking type affect color stability. Consider patient education on smoking cessation.
Effects of cigarette smoking on color stability of dental resin composites^10^ (2017)	An *in vitro* analysis	60 composite resin discs - Durafill YSh, Tetric EvoCeram BulkFiW, and Filtek Supreme Ultrad	Conventional cigarettes (3R4F) and tobacco aerosol (THS2.2)	Cigarette smoke caused significant color changes in composite discs. Tobacco aerosol had milder effects on color. The type of exposure and composite used influenced surface gloss and surface roughness.	Resin composite type and exposure type influence aesthetic changes. Consider treatment planning and material selection.
Effects of cigarette smoke and tobacco heating aerosol on color stability of dental enamel, dentin, and composite resin restorations^16^ (2019)	An *in vitro* analysis	Human premolar teeth with Class V cavities and composite restorations	Conventional cigarettes (3R4F CS) and tobacco heating system aerosol (THS 2.2)	Conventional cigarettes caused significant color changes in teeth and restorations. THS 2.2 aerosol had a significantly lower color impact. Composite restorations were more affected by cigarette smoke.	Different smoking types have varying effects on color stability. Dental ceramics may offer better aesthetics for smokers.
Influence of Electronic Nicotine Delivery Systems (ENDS) in comparison to conventional cigarette color stability of dental restorative materials^17^ (2020)	An *in vitro* analysis	30 composite resin and 30 dental ceramic disk specimens	Conventional cigarettes (Marlboro Gold) and electronic nicotine delivery systems smoke (SMOK - Alien 220w)	Significant color differences in ceramic and composite specimens after exposure to smoke. Cigarette smoke had a slightly lower impact on composite specimens than ENDS aerosol.	Smoking and ENDS aerosol affect the color stability of restorative materials. Consider material selection and patient education.
Staining beverages and cigarette smoke on composite resin and human tooth fluorescence by direct spectrometry^18^ (2017)	An *in vitro* analysis	120 specimens - Four composite resin brands (Microfiller, Nanohybrid, Microhybrid, Nanofiller) and human molars	Staining beverages (coffee solution, cola-based soft drink) and cigarette smoke	Composite resins responded differently to staining agents and cigarette smoke. Different composite resins exhibited variations in fluorescence intensity.	Smoking and staining agents impact color stability differently in various composite resins. Consider material choice and patient education.

**Table 2:** An overview of how ΔE (a measure that plays an important role in evaluating color accuracy) values vary in response to different factors, helping dental professionals understand the impact of smoking types, composite material selection, and the interaction with staining agents on dental restoration aesthetics

*Factors*	*ΔE values*	*Interpretation*
**Smoking types**	Conventional > Electronic	Conventional cigarettes result in more color change, indicating a greater impact on dental aesthetics.
**Composite material selection**	Nano-hybrid > Microhybrid	Nano-hybrid composites are more susceptible to color change, emphasizing the role of material selection.
**Exposure to staining agents**	Smoking + Staining Agents > Smoking alone	Combining smoking with staining agents leads to greater color changes in dental restorations.

**Table 3:** Results for different composite types and their response to various smoking conditions. It shows the mean ΔE values and the associated p-values. Overall, these findings indicate the impact of smoking on the color stability of different composite materials

*AuthorsYear*	*Material used*	*Smoking type*	*Smoking exposure time*	*Mean ΔE*	*SD*	*p*
**Alnasser et al.^15^ 2021**	Nano-hybrid composite	Conventional	90 min	1.714	1.166	0.001
		Electronic		0.646	0.301	0.001
	Micro-hybrid composite	Conventional		0.8512	0.589	0.001
		Electronic		0.487	0.262	0.033
**Zhao et al.^10^ 2017**	DVS	3R4F	20 C/day for 3 weeks	20.7	1.4	<0.0001
		THS2.2		3.3	0.9	<0.0001
	TEC	3R4F		26.2	2.7	<0.0001
		THS2.2		1.9	0.6	<0.0001
	FSU	3R4F		28.0	2.5	<0.0001
		THS2.2		2.6	0.5	<0.0001
**Zanetti et al.^16^ 2019**	Enamel	3R4F	20 C/day, 4 days a week for three weeks	7.1	2.3	<0.0001
		THS2.2		2.0	1.0	<0.0001
	Dentin	3R4F		18.2	3.9	<0.0001
		THS2.2		2.4	0.7	<0.0001
	Composite resin	3R4F		25.6	3.8	<0.0001
		THS2.2		3.0	1.0	<0.0001
**Vohra et al.^17^ 2020**	Dental ceramic	CS	10 puffs per cycle, 10 cycles/day for 7 days	2.422	0.771	<0.001
		ENDS		2.396	0.596	<0.001
		NS		0.291	0.23	Not significant
	Dental composite	CS		42.871	2.448	<0.001
		ENDS		46.866	3.641	<0.001
		NS		0.558	0.329	Not significant

THS: tobacco heating system. DVS: Durafill VS. TEC: Terrie EvoCeram BulkFill. FSU: Filtek Supreme Ultra. CS: cigarette smoke. ENDS: e-cigarette smoke. NS: no smoke. C: cigarettes.

The findings from the selected studies collectively shed light on the aesthetic effects of smoking on resin-based dental restorations, particularly composite resins, and provide valuable insights into how different factors contribute to changes in color stability and other aesthetic properties. These insights have significant implications for clinical practice.

### Type of smoking: conventional cigarettes versus electronic cigarettes

Two included studies, Alnasser et al.^15^ and Vohra et al.^17^ highlighted significant differences in the aesthetic impact of conventional cigarette smoke (3R4F CS) versus electronic cigarette aerosol (ENDS) on resin-based restorations. Conventional cigarettes were found to have a more pronounced effect on color change, resulting in increased yellowness and redness compared to electronic cigarettes. This distinction is essential, as it suggests that conventional cigarettes can have a more detrimental effect on the aesthetics of dental restorations. It is consistent with the general perception of tobacco smoking as being unhealthy: tar and other combustion products from conventional cigarettes deposit on dental restorations, causing them to become discolored^19^. In contrast, electronic cigarettes, which heat a nicotine-containing liquid to produce an aerosol, produce fewer combustion-related substances, resulting in less severe staining effects^14,20^. Dental professionals should educate patients about these differences and emphasize the importance of smoking cessation or switching to less harmful alternatives, especially in cases where the appearance of restorations is a concern.

### Choice of composite material: nano-hybrid versus micro-hybrid composite resins

The studies by Alnasser et al.^15^ and Zhao et al.^10^ underscored that choosing composite material is a critical factor in determining color stability. Exposed to smoking, nano-hybrid composites tended to discolor at a greater rate than micro-hybrid composites. This implies that the nature of these materials significantly affects their susceptibility to stains and shade variations.

The heightened susceptibility of nano-hybrid composites to color changes compared to micro-hybrid composites when exposed to smoking can be attributed to several factors. Nano-hybrid composites contain nanoparticles with larger surface areas, making them more prone to adsorption and chemical reactions with smoking-related compounds^21,22^. Their rougher surface texture, potentially increased porosity, different chemical composition, and adhesion properties further contribute to their vulnerability to staining^23,24^.

This finding emphasizes the significance of selecting the appropriate composite material in dental restorations. Dental professionals should consider the patient’s smoking habits and choose composite materials that are less likely to undergo color changes. This tailored approach can contribute to longer lasting and aesthetically pleasing restorations.

### Exposure to smoking and staining agents

Zhao et al.^10^ and de Silva et al.^18^ have demonstrated the detrimental effects of exposure to staining agents, such as coffee and cola-based soft drinks, on the color stability of composite resins. In combination with smoking, this may exacerbate the aesthetic effects of dental restorations. Patients who smoke and consume staining agents may experience more rapid and noticeable color changes in their restorations.

The worsening of aesthetic impact on dental restorations in patients who smoke and consume staining agents like coffee and cola-based soft drinks can be attributed to the synergistic effects of these factors. The act of smoking introduces substances that cause staining and changes the color of dental restorations. At the same time, staining agents carry molecules with color-producing properties that can attach to dental materials, causing additional discoloration. When these two influences are combined, they mutually reinforce each other, causing faster and more noticeable alterations in color^25^. The composite resins’ porous structure and surface properties can enable the binding of compounds from both smoking and staining agents, making them especially prone to the compounded effects of staining^26^.

This discovery has dual clinical implications. Dental practitioners should educate patients not only about the aesthetic consequences of smoking but also underscore the significance of reducing exposure to staining agents that can interact with smoking-related discoloration. Strategies may involve advising patients to limit the consumption of such beverages or suggesting regular cleaning and maintenance of their restorations to alleviate the effects.

### Dental ceramic versus composite resins

Vohra et al.^17^ compared dental ceramic and composite resin restorations, revealing that composite resins were more susceptible to the effects of conventional and electronic cigarettes. This suggests that ceramic restorations might offer better color stability when patients are smokers.

The observation that dental ceramic restorations provide superior color stability for smokers, in contrast to composite resin restorations, can be ascribed to the inherent characteristics of these materials. Composite resins, generally more porous, are susceptible to discoloration, particularly in the presence of substances like cigarette smoke^25^. In contrast, dental ceramics are well known for their excellent color stability and resistance to staining agents^27^. The variation in material properties enables ceramic restorations to resist the aesthetic impact of smoking better than composite resins.

Although composite resins often yield excellent esthetic results, this study highlights the importance of thoughtful material selection, especially for patients with a smoking history. Dental professionals must weigh the esthetic benefits of composite resin against the risk of discoloration, potentially opting for ceramic restorations in smokers.

### Color change and aesthetic parameters: ΔE values and more

Four reviewed studies used color difference (ΔE) values to measure the degree of color alteration in dental materials, including resin-based dental restorations. The ΔE values are crucial in evaluating the aesthetic impact of different factors. These values provide a standardized and numerical way of indicating the visible color change of a dental restoration, demonstrating the extent to which its color has shifted^28^.

In the context of the discussion, ΔE values are instrumental in comparing the effects of different factors on dental restorations. For instance, when assessing the influence of smoking types, such as conventional cigarettes and electronic cigarettes, ΔE values precisely measure the color change, with higher ΔE values indicating more noticeable shifts. Dental professionals can use these values to understand the extent to which different smoking types affect restorations’ aesthetics^29^. Likewise, when assessing composite material options, ΔE values assist in differentiating between materials with increased or decreased vulnerability to color alterations. This assists dental professionals in choosing the most suitable materials aligned with their patient’s preferences and habits^30^.

When examining the impact of smoking and staining agents like coffee and soft drinks on dental restorations, dentists use ΔE values to measure their combined effects. Higher ΔE values in this context indicate more rapid and noticeable color alterations, signifying that patients who smoke and consume staining agents may experience pronounced changes in the appearance of their restorations^31,32^.

In addition, de Silva et al.^18^ introduced fluorescence intensity as another aesthetic parameter to assess the impact of staining agents and smoking. This expanded perspective highlights how dental restorations’ appearance can be affected and reinforces the importance of considering multiple factors in esthetic evaluations.

### Clinical implications

From the analysis of the studies, we may conclude that the dentist’s awareness of the effect of smoking on resin-based restorations is essential. Dental professionals should be able to distinguish the type of cigarette used as the degree of staining can be influenced by it. Moreover, dentists should educate their patients on smoking cessation or the possibility of switching to less harmful alternatives. In addition, the material selection should be carefully made based on the patient’s smoking habits, as composites tend to stain more easily than ceramic restorations. Therefore, a tailored approach needs to be adopted for individual patients to provide the best results.

Educating patients is crucial in minimizing the impact of smoking and staining agents on dental aesthetics. Dental professionals can enlighten patients on how smoking affects dental restorations and the importance of limiting exposure to staining agents like coffee and soft drinks. Encouraging lifestyle changes that reduce the consumption of these agents can help sustain the aesthetics of dental restorations and promote better oral health. When choosing composite materials for dental restorations, dental professionals should consider the patient’s habits and susceptibility to staining since it is crucial in determining color stability. The study suggests that dental ceramics might offer better color stability for smokers, indicating the potential of alternative materials for patients with a history of smoking. However, composite resins are generally preferred for their esthetic outcomes.

Using aesthetic parameters like ΔE values and fluorescence intensity, highlights the need for a multifaceted evaluation of dental restorations. Dental practitioners should consider these parameters to comprehensively assess the impact of smoking and staining agents.

### Limitations

Limitations of the reviewed studies include methodological variability, such as differences in staining agents, exposure duration, and assessment methods, potentially limiting generalizability and introducing bias. Controlled environments may not fully replicate real-world conditions, affecting external validity. Varied sample sizes and follow-up durations impact robustness, suggesting the need for longer term studies with larger samples.

Since the review was conducted by one author, the interpretation of the literature, selection of included studies, and synthesis of findings may be influenced by the author’s personal perspectives, experiences, and biases. Although a literature search was performed, as the review was not a systematic review, but a narrative review, it is possible that it does not include all studies on the topic. Furthermore, the possibility of publication bias in this aspect was not formally addressed.

Future research should include a systematic review, while original research should focus on longitudinal studies with larger samples, real-world conditions, development of stain-resistant dental materials, assessment of patient profiles, and expanding aesthetic evaluation parameters beyond ΔE values and fluorescence intensity to provide more nuanced insights.

## CONCLUSION

Differentiating the impact of conventional cigarettes and electronic cigarettes on dental restorations is crucial. Conventional cigarettes, due to their combustion byproducts, exhibit more substantial color changes, necessitating patient education on these differences and the importance of smoking cessation or safer alternatives. Material selection should be tailored, with nano-hybrid composites showing susceptibility to color changes in the presence of smoking. Patient education regarding smoking and staining agents is essential to mitigate aesthetic impact. Considering dental ceramics for smokers may provide more stable results. The use of aesthetic parameters beyond ΔE values, such as fluorescence intensity (FI), enhances understanding. While these insights are valuable, limitations exist, and future research should focus on longitudinal, real-world studies and alternative materials. In the evolving field of dental restorations, these findings offer an initial foundation for dental professionals to deliver aesthetically pleasing and durable results, even in the context of smoking and staining agents.

## Data Availability

The data supporting this research are available from the author on reasonable request.
